# Extraction of winding parameters for 33/11 kV, 30 MVA transformer based on finite element method for frequency response modelling

**DOI:** 10.1371/journal.pone.0236409

**Published:** 2020-08-27

**Authors:** Avinash Srikanta Murthy, Norhafiz Azis, Jasronita Jasni, Mohammad Lutfi Othman, Mohd Fairouz Mohd Yousof, Mohd Aizam Talib

**Affiliations:** 1 Advanced Lightning, Power and Energy Research Centre (ALPER), Department of Electrical and Electronic Engineering, Faculty of Engineering, Universiti Putra Malaysia, Serdang, Selangor, Malaysia; 2 Institute of Advanced Technology (ITMA), Universiti Putra Malaysia, Serdang, Selangor, Malaysia; 3 Faculty of Electrical and Electronics Engineering, Universiti Tun Hussein Onn Malaysia, Parit Raja, Johor, Malaysia; 4 TNB Research Sdn. Bhd., Kawasan Institut Penyelidikan, Kajang, Selangor, Malaysia; University of Vigo, SPAIN

## Abstract

This paper proposes an alternative approach to extract transformer’s winding parameters of resistance (R), inductance (L), capacitance (C) and conductance (G) based on Finite Element Method (FEM). The capacitance and conductance were computed based on Fast Multiple Method (FMM) and Method of Moment (MoM) through quasi-electrostatics approach. The AC resistances and inductances were computed based on MoM through quasi-magnetostatics approach. Maxwell's equations were used to compute the DC resistances and inductances. Based on the FEM computed parameters, the frequency response of the winding was obtained through the Bode plot function. The simulated frequency response by FEM model was compared with the simulated frequency response based on the Multi-conductor Transmission Line (MTL) model and the measured frequency response of a 33/11 kV, 30 MVA transformer. The statistical indices such as Root Mean Square Error (RMSE) and Absolute Sum of Logarithmic Error (ASLE) were used to analyze the performance of the proposed FEM model. It is found that the simulated frequency response by FEM model is quite close to measured frequency response at low and mid frequency regions as compared to simulated frequency response by MTL model based on RMSE and ASLE analysis.

## Introduction

Transformers are one of the important components in the electrical distribution network. The continuity of the energy delivery depends on the reliable operation of transformers. The health of transformers can be affected by several factors such as degradation of insulations, axial/radial displacements and mechanical deformation of windings. Furthermore, high electromechanical forces due to short circuits could exacerbate the structural integrity of transformers. Condition assessment through non-intrusive method such as Frequency Response Analysis (FRA) could provide early warnings for mitigation purpose which in turn could reduce any unexpected failures of transformers.

FRA is known as an off-line measurement technique that can be used to detect mechanical integrities of transformer windings [1–4]. Generally, FRA of transformers is measured during factory acceptance test for baseline fingerprint and before site installation in order to ensure the winding structures are intact during transportation process. The transformer’s winding can be represented through the electrical equivalent circuit, which consists of resistance (R), inductance (L), capacitance (C) and conductance (G). The electrical equivalent circuit is normally represented by several sets of RLCG matrices. The matrix R is known as the winding conductor skin resistance. The L matrix consists of self and mutual inductances. The C matrix consists of *C*_*t*_ (turn-to-turn capacitance), *C*_*d*_ (inter-disc capacitance) and *C*_*g*_ (capacitance between winding conductor and ground). The G matrix consists of the conductor conductivity due to the insulation loss. These parameters can be calculated based on the geometrical specification of the winding structure such as size of the conductor, number of turns/discs and the thickness of the Kraft paper insulation. The RLC parameters of windings normally could be affected if there is an internal mechanical change. FRA can be a good tool to determine the winding’s condition in-service through comparison between in-service and baseline fingerprint frequency responses. If there is no baseline fingerprint, comparison of frequency responses among the phases could provide indication on any mechanical deformations/movements of the windings. In addition, sufficient database and historical information could support to pinpoint the corresponding faults related to the deformations and movements of the windings.

Among the common analytical models used to evaluate the FRA in transformers are high-frequency single input and multiple output circuit model based on Multiconductor Transmission Line (MTL), duality-based lumped circuit model and high frequency lumped circuit model that consider inter-turn capacitance as well as mutual inductance between the High Voltage (HV) and Low Voltage (LV) winding conductors [5,6]. Other model uses the travelling wave equation for modeling the winding discs coupled with MTL model [7]. The frequency response has also been modeled by Fourier transform technique for excitation and response signals study [8]. One of the key parameters for FRA modeling for transformer windings is the RLC parameters. These parameters are mainly computed based on analytical techniques that considers cascaded pi-network and diamagnetic effect of the winding [9–11].

The calculations of the RLCG parameters are extensive once the apparent damage is detected in transformers based on the MTL and lumped circuit approaches [12]. Furthermore, the RLCG parameters are estimated values due to several assumptions and simplifications. Other study has used Finite Element Method (FEM) in order to extract the detail information of the RLCG parameters [13–15]. FEM is computationally extensive whereby normally only specific sections of the windings are computed [16,17]. Furthermore, the computation of RLCG parameters is a complicated procedure due to consideration on multiple parameters [18]. The comparison between MTL, lumped circuit and FEM can be seen in Table 1. The complexity of FRA/overvoltage application is comprehensible and quite detail in comparison with MTL and lumped circuits models. FEM model offers viable option to examine the impact of individual structural damages of the windings which can be costly to be carried out through experimental approach [19]. However, considering the time-consuming factor for FEM, there is a need to improve the approach and at the same time provides acceptable simulated frequency response.

**Table 1:** Comparison between MTL, lumped circuit and FEM approaches.

Parameters	MTL	Lumped Circuit	FEM
Geometry design complexity	High	High	High
Model representation	Numerical approach	Electrical parameters in the circuit	3-D model
RLC extraction	Analytical	Analytical	Numerical field analysis
Overvoltage transient analysis / FRA	Depends on representation of model elements	Depends on representation of model elements	Same model for both FRA and overvoltage analysis
Application Complexity	Mid	High	Low
Simulation time for frequency response	Mid	Low	High

This paper proposes a FEM based model for the 33/11 kV, 30 MVA disc type transformer winding to generate the RLC parameters for FRA purpose. The RLCG parameters are extracted through the quasi-state Ansys Q3D platform based on the Method of Moment (MoM) and Fast Multiple Method (FMM). The extracted RLC parameters are exported to Ansys Simplorer circuit to simulate frequency response.

The novelty of this research work is the procedure that utilize several algorithms in a systematic manner in order to extract the RLCG parameters and generate the FRA plot through quasi-state Ansys Q3D and Simplorer platforms. The main aim of the research work is to develop a solver scheme based on the FEM platform for analyzing the transformer’s winding deformation. The proposed FEM based model can be used for both structural and overvoltage behavior of the winding structure. The RLC parameters are also extracted through analytical method and the frequency response is simulated based on the Multi-conductor Transmission Line (MTL). The simulated frequency response based on FEM is compared with simulated frequency response obtained from MTL and measured frequency response.

## Methodology

**Fig 1:**
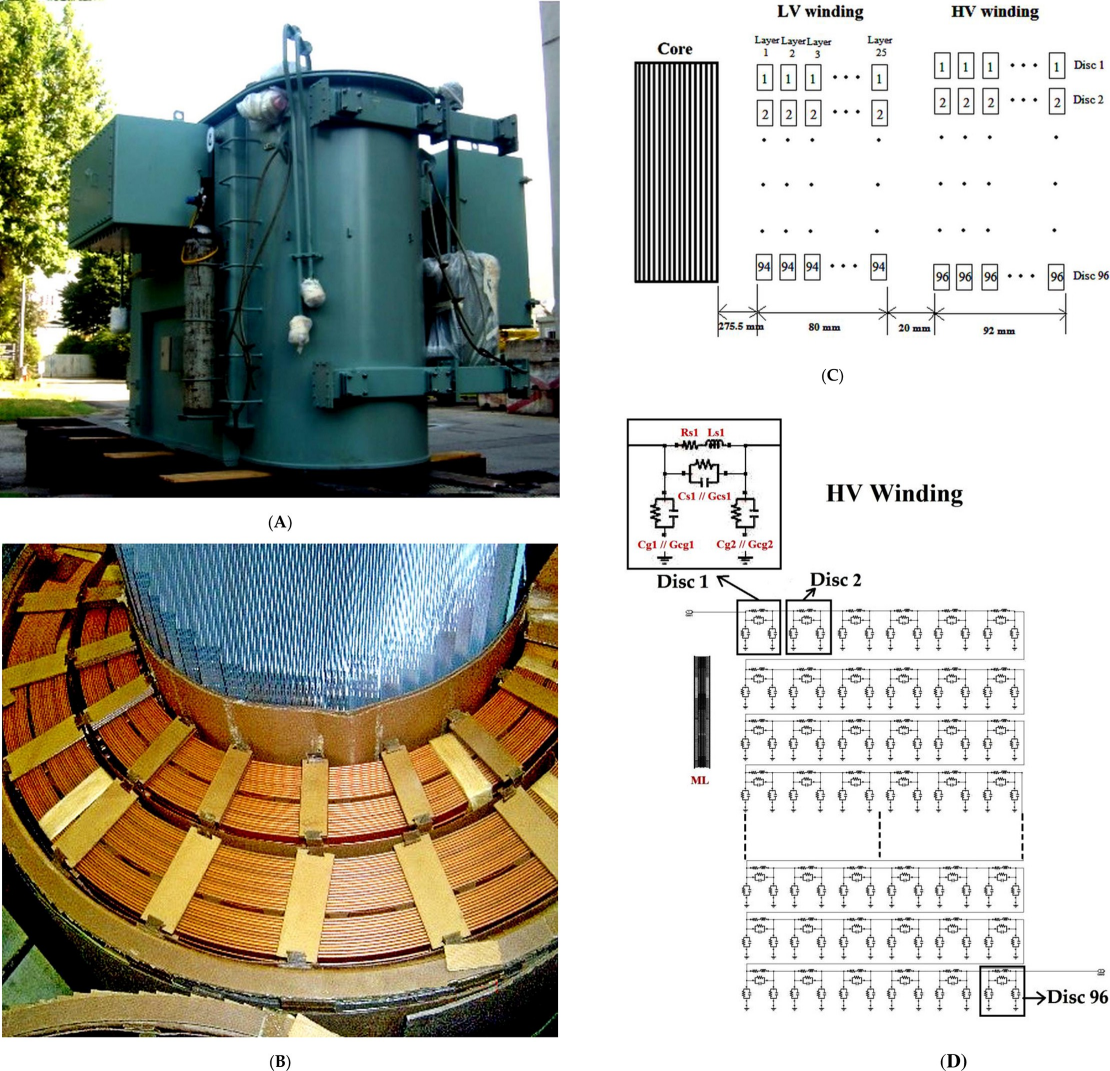
(A) Side view. (B) top view of the HV and LV windings. (C) cross-sectional view for the LV and HV windings. (D) RLC equivalent circuit for the HV winding of the 33/11 kV, 30 MVA transformer.

A disc layered HV winding of a Dyn11 33/11 kV, 30 MVA transformer was investigated in the study. The side view of the transformer under study can be seen in Fig 1A. The top view of HV and LV winding can be seen in Fig 1B The cross-sectional view for the single-phase winding can be seen in Fig 1C. The RLC equivalent circuit of the single phase HV winding can be seen in Fig 1D. The geometry specifications for HV winding are shown in Table 2. The HV winding of a single phase consists of 96 discs and each of the discs has 6 conductors with 5 turns. The insulation thickness between each of the conductors is 0.5 mm. There is a cooling duct with 5 mm thickness between turns 12 and 13. The distance between each of the discs is 3 mm. The distance between HV and LV winding is 20 mm. The inner and outer radiuses of the HV winding are 374.5 mm and 462.5 mm respectively. Each of the sections is represented by a disc whereby the lumped RLC parameters of the HV winding can be seen in Fig 1D. *R*_*s*_ is the series resistance, *L*_*s*_ and *M*_*L*_ are the self and mutual inductance of each disc. *R*_*s*_ and *L*_*s*_ are connected in series. *C*_*s*_ and *G*_*cs*_ are the total series capacitance and conductance that are connected in parallel. *C*_*g*_ and *G*_*cg*_ are the ground capacitance and conductance between ground and winding conductor that are connected in parallel.

**Table 2:** HV winding geometry specification of the 33/11 kV, 30 MVA transformer.

Parameters	Values
Height of the conductor	11.5 mm
Width of the conductor	2.4 mm
Thickness of the insulation (double-sided)	0.5 mm
Number of turns per disc	30
Number of discs in one phase	96
Distance between each of the discs	3 mm
Cooling duct between turns 12 and 13	5 mm
Inner radius of the HV winding	374.5 mm
Outer radius of the HV winding	466.5 mm
Total circumference of the HV winding	79.17 m
Height of HV winding	1437 mm
Insulation between HV–LV windings (9 mm of Oil + 1 mm of pressboard)	20 mm
Relative permittivity of the insulation, *ε_r_*	2.3

**Fig 2:**
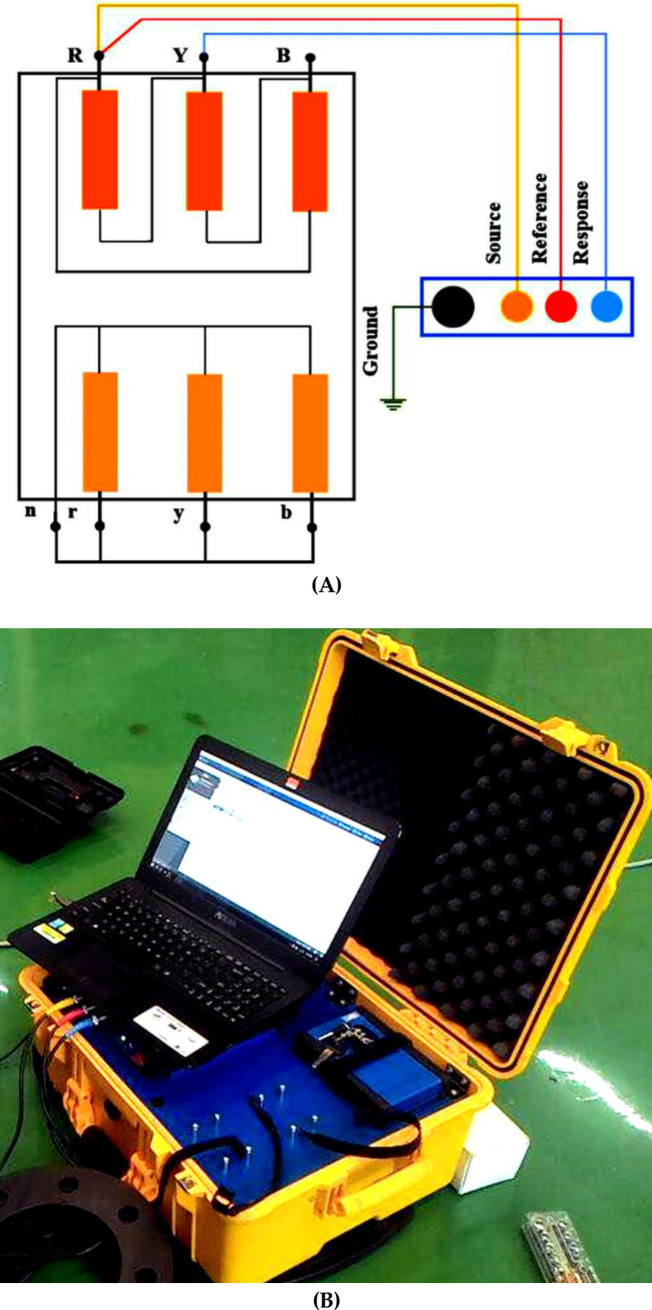
FRA measurement configurations: (A) end-to-end short circuit HV. (B) FRA analyzer.

There are 4 test connections to perform the FRA measurement based on CIGRE WG A2/26 [19], IEEE Std C57.149–2012 [4] and IEC 60076–18 [20]. In this study, only end-to-end short circuit test was conducted for HV winding according to IEEE Std C57.149–2012 [4]. The end-to-end short circuit was measured at Y phase of the HV winding through the injection of a 10 V AC input signal at frequencies from 20 Hz to 2 MHz at R phase of the HV winding as shown in Fig 2A. The B phase of the HV winding was left open and all 3 LV winding terminals were short-circuited to generate the frequency response of a single phase. The FRA was measured through OMICRON FRAnalyzer FRANEO 800, as shown in Fig 2B.

### A. Modeling of the frequency response based on MTL method

**Fig 3:**
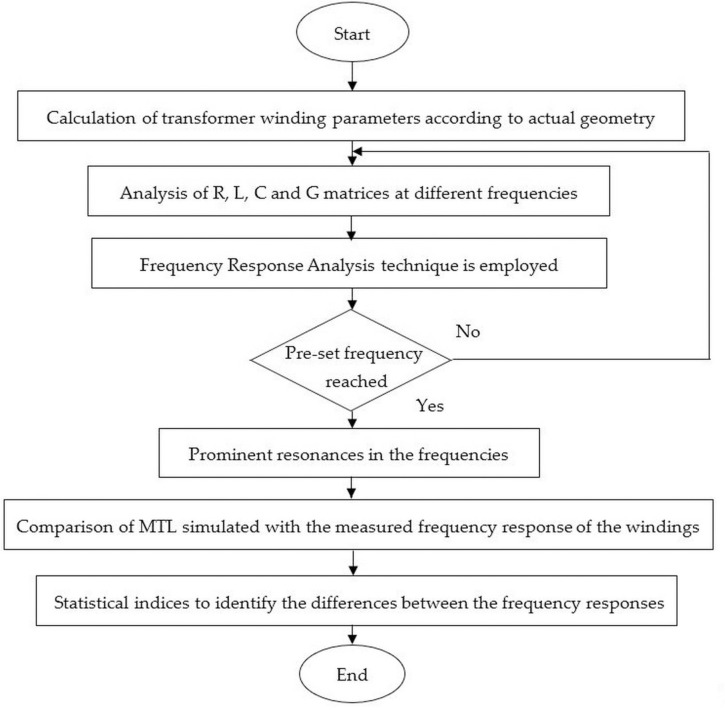
Procedure for simulated frequency response based on MTL model.

The procedure to simulate the frequency response of the transformer winding based on MTL model can be seen in Fig 3. The RLCG parameters were obtained based on analytical equations, which considers the actual geometrical specifications of the winding through MATLAB platform. The RLCG parameter matrices were analyzed at different frequencies up to 2 MHz. Next, the frequency response was simulated based on MTL model according to end-to-end short-circuit FRA measurement. The prominent resonances in the frequencies were identified. The simulated frequency response based on MTL model was compared with the measured frequency response. The final step was to carry out the statistical analysis to identify the differences between simulated and measured frequency response.

The electrical parameters such as inter-turn/inter-disc capacitances, self/mutual inductances, resistance and conductance were calculated based on the height and width of the conductor, number of turns in the disc, thickness of the insulation, distance between turns and discs, distance from the centre of the core to the inner and outer radius, height and total circumference of the HV winding structure. The geometrical details of the HV winding can be seen in Table 2. The self and mutual inductances were calculated, and inductance matrix was constructed by taking into account the eddy current losses in the core and windings based on equations in the earlier studies [21–24]. The diagonal and off-diagonal elements were set as the self and mutual inductances of the inductance matrix having length of 96 × 96. The turn-to-turn capacitance, *C*_*t*_ between 2 adjacent conductors of a disc and the inter-disc capacitance, *C*_*d*_ between the adjacent discs were calculated based on equations in [21,22]. *C*_*t*_ between any two conductor was not the same due to the differences on the conductor length of each turn, the total inter-turn capacitance, *C*_*t*,*total*_ and the total inter-disc capacitance, *C*_*d*,*total*_ were calculated based on equations in [21,24]. Since the tank was not grounded during the FRA measurement, the ground capacitance, *C*_*g*_ was neglected in the current calculation. The total series capacitance of one disc, *C*_*s*_ was calculated based on equations in [24] using *C*_*t*,*total*_ and the resultant inter-disc capacitance, *C*_*d*,*t*_ of the whole winding [24]. Due to small core magnetic effect for frequency above 10 MHz, the losses due to the core were not considered in the current calculation [25]. The skin and proximity effects of the conductor were considered during the calculation of series resistance of the HV winding based on equations in [21,22,26]. The conductance, *G* was calculated based on equations in [21,24].

**Fig 4:**
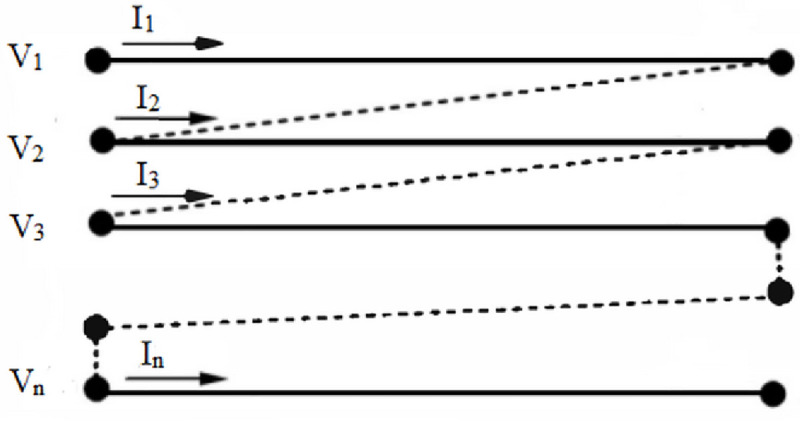
MTL of a winding of a transformer having input and output V-I.

Cascaded pi (Π) network of transmission line was considered in the MTL model where each half of the total line capacitances was connected to the sending and receiving end. This model can be subjected to different types of winding deformations and it can represent as arrangement of conductors in the windings [27]. Based on Very Fast Transient Overvoltages (VFTOs) study, MTL model could solve the transfer function for the FRA by considering transformer windings as single input multiple output model [5,28–35]. The voltage and current were calculated in the frequency domain after the construction impedance and admittance matrices based on [33]. The admittance matrix for the two-port network based on Fig 4 have sending and receiving voltages [33]. The input and output voltages of the winding including 50 ohms cable were considered in the frequency response simulation. The 50 ohms cable was chosen since it was the rated input impedance of the measuring equipment [24,36]. The simulation of the MTL model has been carried according to [9,27,37]. The calculated RLC parameters for MTL model can be seen in Table 3.

**Table 3:** RLC parameters of the HV winding for the 33/11 kV, 30 MVA transformer.

Parameter	Values
Turn-to-turn capacitance, *C*_*t*_	33.5 pF
Total inter-disc capacitance, *C*_*d*_	526.89 μF
Series capacitance, *C*_*s*_	208.68 μF
Series Resistance, *R*_*s*_	4.67 Ω
Conductance *G*_*s*_	131.12 μΩ
Inductance, L	96×96

### B. Modeling of the frequency response based on the MoM and FEM methods

**Fig 5:**
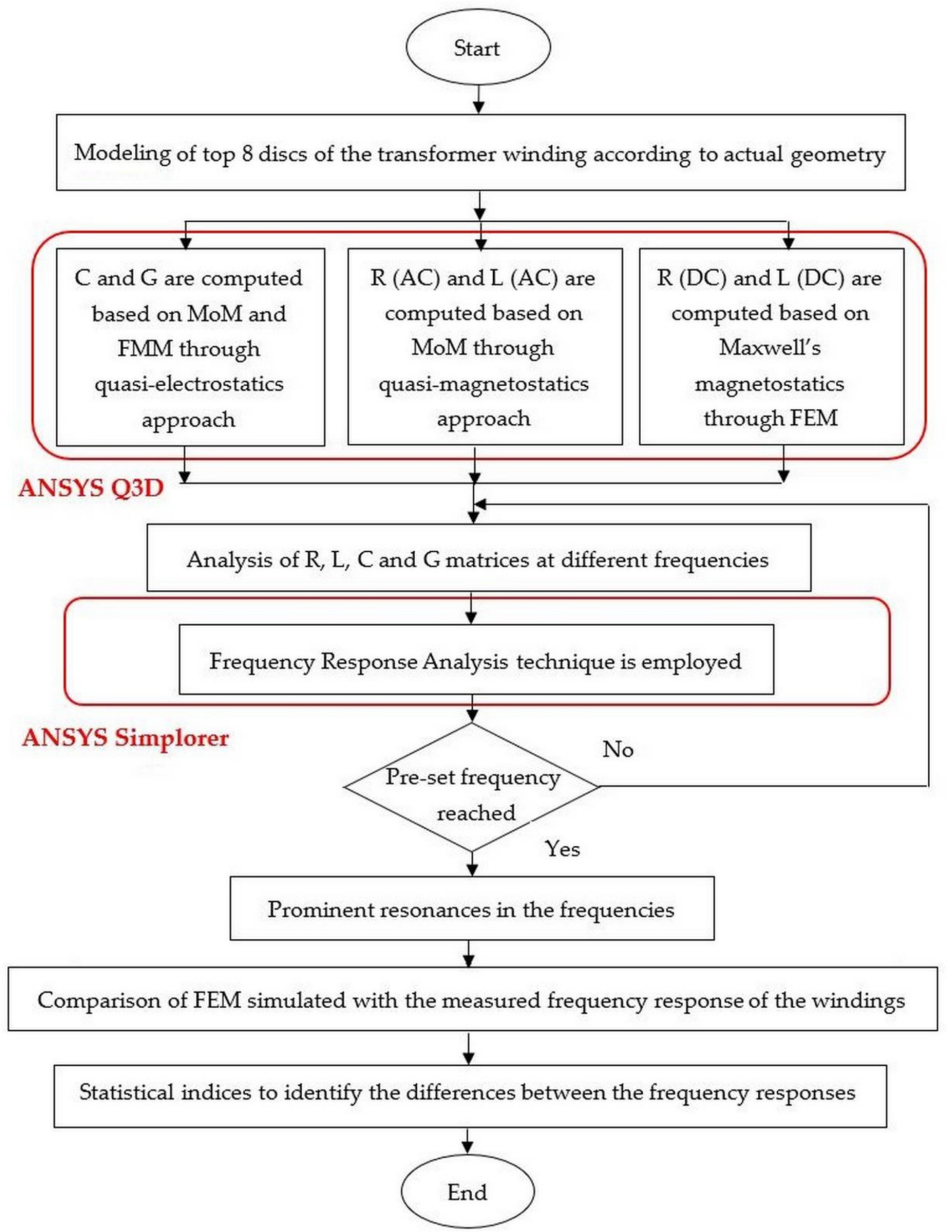
Procedure for simulated frequency response based on FEM.

The procedure to obtain simulated frequency response of the transformer winding based on FEM can be seen in Fig 5. The transformer winding of 6 conductors, 5 turns and 8 discs was modeled based on actual geometrical specifications. The capacitance and conductance were calculated based on MoM and FMM methods through quasi-electrostatic approach. AC resistance and inductance were calculated based on MoM through quasi-magnetostatic approach. DC resistance and inductance were computed based on Maxwell’s magnetostatic approach based on FEM. All these winding parameters were computed in ANSYS Q3D platform. The computed RLCG parameter matrices were analyzed at different frequencies. Next, the extracted parameters were extracted into ANSYS Simplorer for the simulation of frequency response. The prominent resonances were identified, and the simulated frequency response based on FEM was compared with the measured frequency response. The statistical indices were used to identify the differences between simulated frequency response by FEM and measurement.

During the electromagnetic computation of the parameters of the transformer winding, the parasitic components and conduction paths that are non-ideal in nature were considered. The R, L, C and G of the winding 3D structure were calculated including the self and mutual elements for each of the components. The C, G, AC resistance and AC inductance were computed through MoM and Fast Multipole Method (FMM) [38]. The DC resistance and DC inductance were calculated based on FEM through current volume density approach of Maxwell’s equations.

The passive components cannot be considered as ideal and the same rule was also applied to all conductors such as traces, vias and ground planes that provide a current path on the transformer winding. The non-ideality of the result in small and often unwanted impedances called parasitic components that are distributed across the entire winding. Since the transformer winding model was considered in the open environment, the infinite ground plane was set as the default boundary condition for parameter extraction in Ansys Q3D Solver.

#### a. The calculation of capacitance

The calculation of capacitance was carried out based on the MoM [38,39]. This approach is more efficient than Maxwell equations for extraction of capacitances since it uses the discretization of boundary conditions through quasi-electrostatics [40]. The capacitance of the disc winding includes turn-to-turn and inter-disc capacitances. The turn-to-turn capacitances for each of the turns were calculated according to equations in [9,21,24,41]. The mutual capacitance, i.e., capacitive coupling between the conductors for each of the discs was taken into consideration due to the presence of the conduction paths in the time-varying electric field. The electrical fields between the two conductors are due to the difference in voltage potential between two winding conductors. The mutual capacitance between two conductors exists once there is a conduction path for Electromagnetic Interference (EMI) [38,39]. The magnitude of the voltage induced into the remaining conductors depends on the input voltage on the first conductor, *V*_*s*_(*t*) and mutual capacitance, *C*_*m*_. The noise is generated when the voltage is induced at the sink conductors. The noise in the voltage was calculated based on Eqs (1) and (2) [38,39,41]:

$$V_{n}\left(t\right)=j\omega R_{l}C_{m}V_{s}\left(t\right)\;\mathrm{when}\;R_{l}\ll \frac{1}{j\omega \left(C_{v}+C_{m}\right)}$$ (1)

$$V_{n}\left(t\right)=\left(\frac{C_{m}}{C_{v}+C_{m}}\right)V_{s}\left(t\right)\;\mathrm{when}\;R_{l}\gg \frac{1}{j\omega \left(C_{v}+C_{m}\right)}$$ (2)

where *C*_*v*_ is the noise capacitance and *R*_*l*_ is the load resistance in the winding model. The mutual capacitance between two parallel conductors *C*_*m*_ can be calculated based on Eq (3):

$$C_{m}=\frac{27.6\times l\times \varepsilon_{r}}{10^{12}ln\left(\frac{d}{w}+\sqrt{1-{\left(\frac{2w}{d}\right)}^{2}}\right)}F$$ (3)

where *d* is the distance between each disc, *l* and *w* are the length and width of the conductor.

The conductance and capacitance of the winding model were calculated in the quasi-electrostatic setup through Ansys Q3D parameter extractor. Each of the winding conductors was assigned a net of 10 V to one conductor and 0 V to remaining conductors for the computation of parasitic components which was then implemented for meshing of conductors and the insulation. The charges are bound by the multiple boundary conditions that were summed together for each of the layer conductors to obtain a matrix of charge relations between each defined net. The conductance and capacitance between the nets were computed by taking insulation into consideration and the charge, *Q* can be calculated based on equations in [38,39,41]. The charge distribution between each net on the insulation surface was calculated whereby the sum of free charges on each of the conductors contributed to the formation of the matrix was computed by taking charges on the boundary into consideration. The quasi-electrostatics solution results in admittance matrix, [*Y*] = [*G*] + *jω*[*C*] that consists of conductance [*G*] and capacitance [*C*] matrices. The procedure for the construction of matrix C is discussed in [42,43].

#### b. The calculation of AC inductances and resistances based on MoM

The self-inductance of each conductor was calculated based on Eq (4) [38,39,44]:

$$L_{\mathrm{self}}=\frac{0.002l\left[ln\left(\frac{2l}{w+h}\right)+0.5+02235\left(\frac{w+h}{l}\right)\right]}{10^{6}}$$ (4)

where *l* is the length of the conductor, *h* and *w* are the height and width of the winding conductor. The mutual inductance *L*_*m*_ between 2 conductor elements exists once one of the conductors interact with another conductor through a time-varying magnetic field through quasi-magnetostatic [40]. The first conductor is referred to as a source and another as a victim. The impedance matrix would be in the form of *Z* = *R* + *jωL*. The voltage induced in the victim conductor was calculated based on Eq (5). The mutual inductance is the coupling of source conductor with the victim conductor through the magnetic field generated by the varying current is *i*_*s*_(*t*) of source conductor. It was calculated based on Eq (6) [38,42].

$$V_{n}=-L_{m}\frac{di_{s}(t)}{dt}$$ (5)

$$L_{m}=k\sqrt{L_{self,source}\times L_{self,victim}}$$ (6)

where *L*_*self*,*source*_, and *L*_*self*,*victim*_ are the self-inductances of the source and victim conductors and *k* is the coupling factor that would be in between 0 and 1 where the value of 1 indicates a maximum mutual coupling between the inductances of 2 conductors. The value of *k* will also be 1 if there is a reduced magnetic leakage flux between the 2 conductors.

The magnitude of the inductance would be dependent upon the value of the coupling factor and the length of each of the conductors and it was calculated based on Eq (7) [41]:

$$L_{m}=\frac{0.002l\left[ln\left(\frac{2l}{d}\right)-1+\left(\frac{d}{l}\right)\right]}{10^{6}}H$$ (7)

where *l* is the length of the conductor and *d* is the distance between 2 conductors. The effect of the core on the resistance was quite low and hence it had been neglected in the present study [45]. The resistances are frequency-dependent due to the consideration of skin and proximity effects and it can be calculated based on Eq (8) [38,39]:

$$R=\frac{l}{2\sigma \delta \left(h+w\right)}\Omega \;\mathrm{where}\;\delta =\frac{1}{\sqrt{2\pi f\mu_{0}\sigma }}$$ (8)

where *l* is the length of the conductor, *h* and *w* are the height and width of the conductor, *σ* is the conductivity of the conductor, *δ* is the frequency related skin depth, *f* is the frequency and *μ*_*0*_ is the permeability of free space (4*π*×10^−7^). The quasi-magnetostatic setup was used for the computation of impedance matrix with the current excitation in Ansys Q3D parameter extractor.

#### c. The calculation of DC inductance and resistance based on Maxwell equations through FEM

The elements of the DC impedance matrix were computed based on Maxwell equations through Ansys Q3D. The self/mutual inductances and resistances were calculated based on the application of current in the conductor through current volume density approach [46]. The magnetic flux density, B was computed based on electromagnetic field equations [47]. The Ampere’s law for the transient model was computed based on [48,49]. The magnetic energy, W in the conductor was computed based on equations in [9,24,49]. The magnetic current density was obtained through approach in [48]. The self and the mutual impedances were calculated based on previous equations in [24,50].

**Fig 6:**
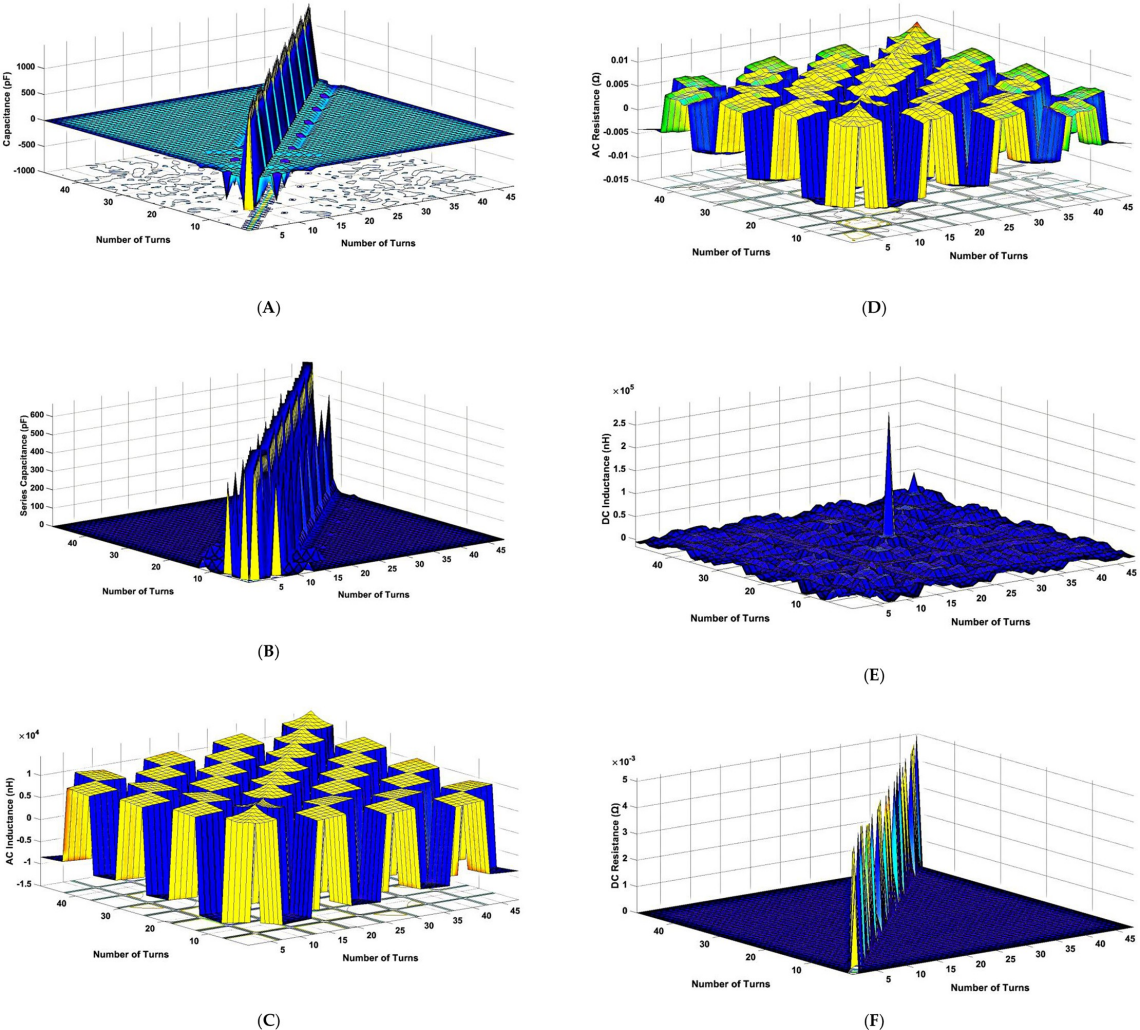
RLC parameters of the 33/11 kV, 30MVA transformer based on Ansys Q3D: (A) capacitance; (B) spice capacitance; (C) AC inductance; (D) AC resistance; (E) DC inductance; (F) DC resistance matrices.

The RLC parameters were computed and the resultant 48 × 48 matrices of the parameters were represented through 3-D Surface-Contour plot that can be seen in Fig 6A–6F. Both capacitance and inductance affect the low-frequency region around the first resonance peak and mid-frequency region in a wide range of the FRA plot. The distribution of the turn-to-turn capacitance, spice capacitance, AC self/mutual inductance, AC resistance, DC self/mutual inductance and DC resistance matrices of the 6 conductors with 5 turns for all 8 discs can be seen in Fig 6A–6F. The diagonal elements in Fig 6A and 6B are defined as the capacitances for conductor 1 for disc 1, conductor 2 for disc 2 and so on. The spice (stray) capacitances also known as the parasitic capacitances were included for consideration on the current circulation along the path of the conductors. The off-diagonal elements are the capacitances between main conductor and its neighbouring conductors. The presence of the inter-turn capacitances next to main diagonal elements (spikes) is due to the close distance between the main and neighbouring conductors.

#### d. Simulation of the frequency response based on FEM

The diagonal and off-diagonal elements formed the self and mutual inductances and resistances of the DC impedance matrix. The equations governing MoM and FEM were computed based on FEM using Ansys Q3D. An automated volumetric adaptive meshing and remeshing with multithreading capability in Ansys Q3D were selected to obtain the conditioned matrix for the RLCG parameters. The convergence percentage error and percentage refinement per pass were set to 1% and 35%. The total number of nodes and elements are 926,167 and 369,168 with minimum edge length of 2.8263×10^−2^ mm. Next, an EMI-favourable layout was setup whereby the R,L,C and G parameters of the transformer’s winding 3D model were employed for the parameters extraction in FEM based Ansys Q3D.

**Fig 7:**
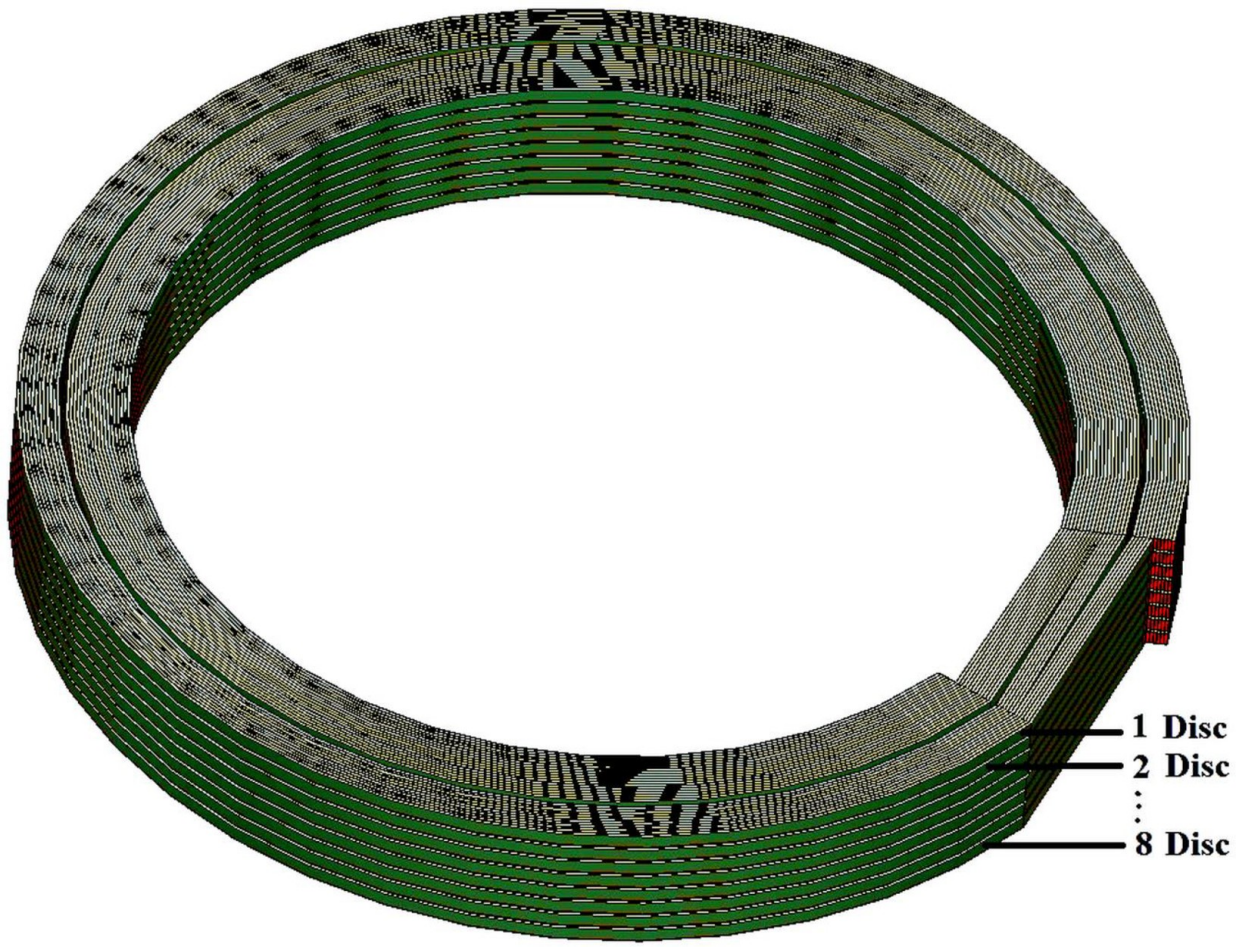
An 8-disc model of the transformer winding.

In order to reduce the FEM computational time, only 8 discs of 30 turns each were modeled for the RLCG parameters extraction based on Ansys Q3D. In addition, each of the discs was not connected in the 3-D model in order to reduce the complexity and computational times as seen in Fig 7.

**Fig 8:**
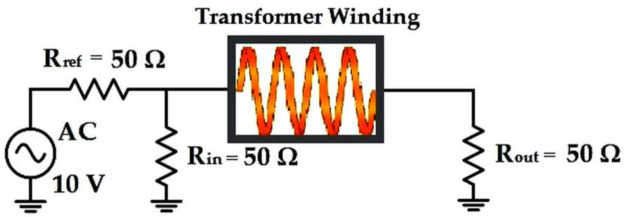
External circuit coupled with a 3-D winding model.

The previous computed electromagnetic properties were utilized alongside the governing equations. Therefore, the transformer’s winding was treated as an electrical component which was connected to the voltage source and co-axial cables to form an FRA measurement circuit. In the current case, Ansys Q3D model with extracted RLCG parameters was combined with Ansys Simplorer FRA circuit that consists of Rref, Rin and Rout. These resistances were set to 50 ohms to compensate for the impedance of the FRA measurement co-axial cable. An external circuit coupled with the 3-D model can be seen in Fig 8.

The frequency responses were obtained based on Eq (9) through injection of low voltage sinusoidal signal *V*_*in*_(*f*) at one end of the terminal and measuring the output voltage *V*_*out*_(*f*) at the other end of the terminal through the resistance connected in series. It should be considered that the source cannot be the same as the current density due to the influence of the winding and asymmetrical peak in current circulation:

$$Magnitude\;in\;dB=20\;\log_{10}\frac{V_{out}\left(f\right)}{V_{in\left(f\right)}}$$ (9)

**Fig 9:**
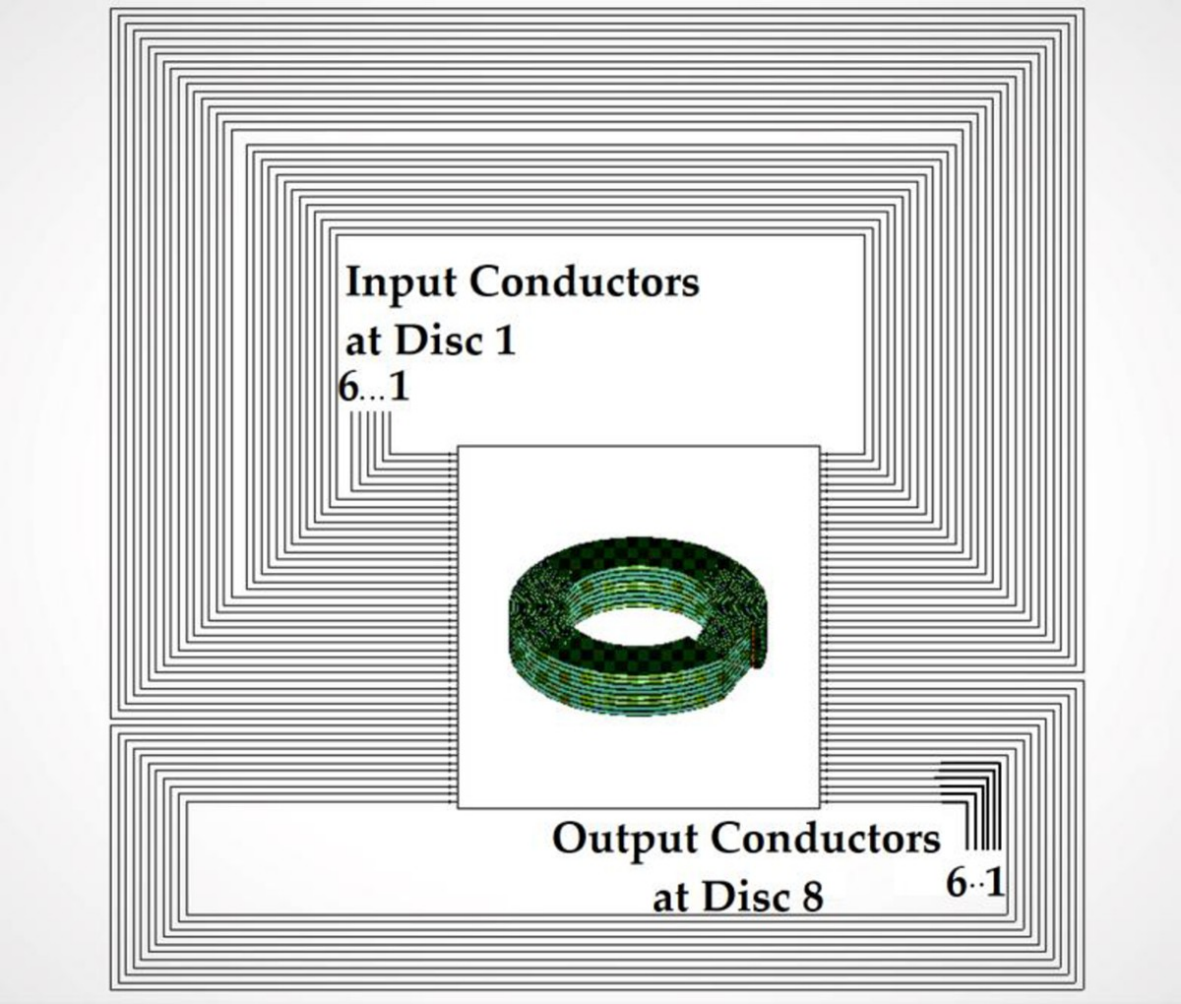
A winding Ansys Q3D model coupled with the Ansys Simplorer circuit for 8 discs.

The FEM model of 8 discs with extracted parameters was imported to Ansys Simplorer for the generation of the frequency response as seen in Fig 9. The model of the transformer winding was designed according to the actual geometry of the transformer windings. The quasi-state Ansys Q3D was used throughout the analysis, from the initial modeling stage where the winding model was modeled, to high-frequency dependent computations. The electrical parameters RLCG were computed based on the assumption of the healthy condition of the transformer to obtain the frequency response.

The method used in the study considered the EMF distribution in the FEM model based on the different connection modes through the variation of the circuit load. Since the proposed model could be used for overvoltage analysis, the properties of copper, steel and insulation material were neglected in the modeling stage [51].

**Fig 10:**
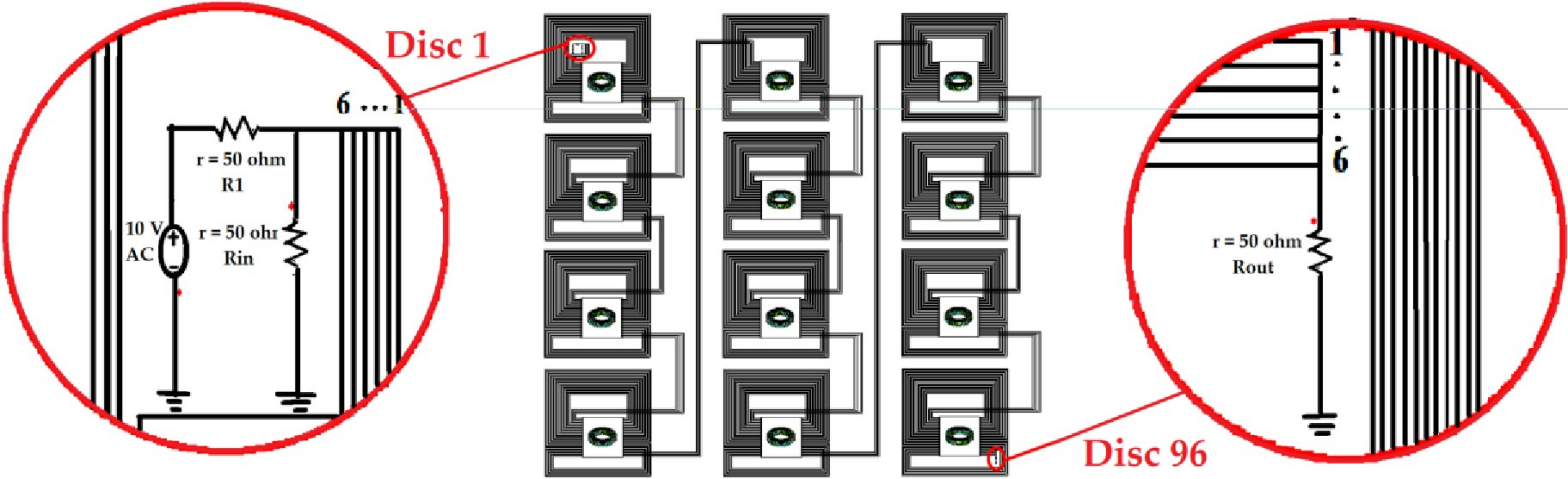
Ansys Q3D coupled model with the Ansys Simplorer circuit for 96 discs to obtain the frequency response.

The Ansys Q3D winding model of 8 discs with extracted parameters was stacked 12 times to form the complete HV winding of a single phase that consists of whole 96 discs can be seen in Fig 10. The winding model with the extracted RLC parameters was treated as an electrical component in FRA equivalent circuit through Ansys Simplorer. The AC voltage of 10 V was applied to 6 conductors of disc 1 of which the measurement was conducted at the 6 conductors of disc 96 for each frequency point from 20 Hz to 2 MHz based on IEEE Std C57.149–2012 [4] as seen in Fig 10. The ratio of voltage across *R*_*out*_ to the voltage across *R*_*in*_ was measured to simulate the frequency response.

#### e. Statistical indices

Statistical methods such as Absolute Sum of Logarithmic Error (ASLE) and Root Mean Square Error (RMSE) were used for the estimation of error with simulated frequency response by MTL and FEM models in comparison with the measured frequency response. It is known that ASLE determine the magnitude difference between two points in the same frequency that has similar slope with different magnitude. ASLE estimates the correlation of 2 dataset variables based on similarity in shapes and not taking magnitudes in consideration [51]. However, RMSE reduces the variance of error distribution. These methods were proposed by standards such as CIGRE WG A2.26 and IEEE Std C59.149–2012 [4,19]. In order to use the statistical indicators, the frequency range of the response needs to be divided into 3 frequency bands: low, medium and high. The frequency range for each of the bands considered in this study was based on the measured frequency response of the transformer winding.

1. Root Mean Square Error (RMSE)

RMSE is the measure of the differences between regression data points that can be determined based on Eq (10) [52–54]:

$$RMSE=\sqrt{\frac{\sum_{i=1}^{N}{\left(X\left(i\right)-Y\left(i\right)\right)}^{2}}{N-1}}$$ (10)

where *N* is the total number of points in two dataset variables and *X*(*i*) and *Y*(*i*) are the *i*-th value of the two dataset variables *X* and *Y*. The value of RMSE should be close to 0 if the 2 dataset variables have a good correlation.

1. Absolute Sum of Logarithmic Error (ASLE)
  ASLE compares the magnitude of two dataset variables in logarithmic scale which includes the property of mean square error (MSE) and can be determined based on Eq (11) [54–56]:
  $$ASLE_{\left(X,Y\right)}=\frac{\sum_{i=1}^{N}\left|20log_{10}Y_{i}-20log_{10}X_{i}\right|}{N}$$ (11)
  where *X*_*i*_ and *Y*_*i*_ are the *i*-th value of the two dataset variables *x* and *y*. The ASLE output should be close to 0 if two dataset variables are similar.

## Results

### A. Comparison between frequency response based on MTL model and measurement

**Fig 11:**
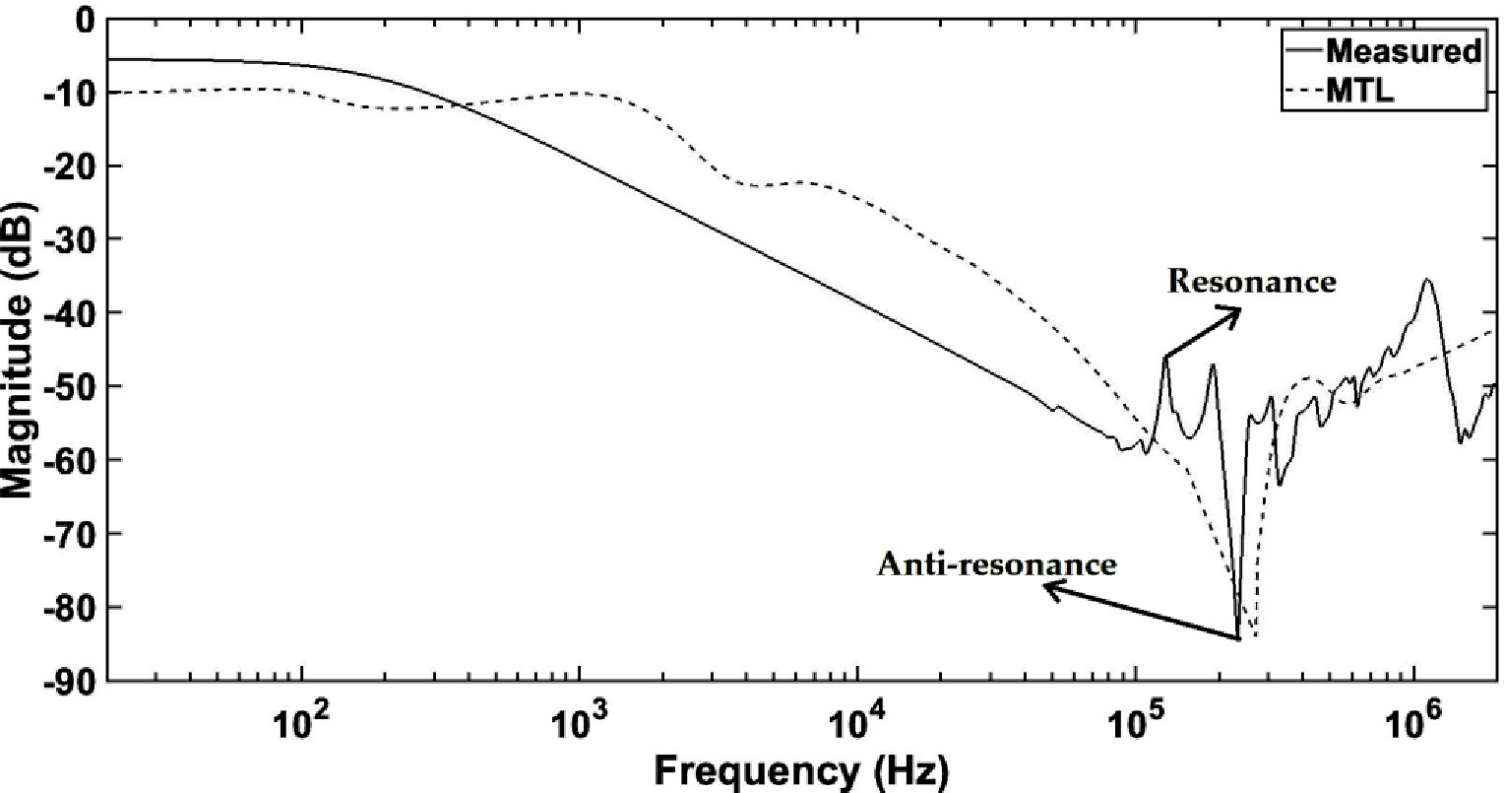
The comparison of simulated frequency response based on MTL model and measurement.

Generally, the curve of simulated frequency response by MTL model follows the measured frequency responses as shown in Fig 11. The measured frequency response has 2 apparent resonances in the mid frequency region due to the interaction of capacitance with the magnetizing inductance of the winding [2]. The occurrence of the first resonance for the measured frequency response can vary with the residual magnetization of the core. Minor resonances occur in the measured frequency response at the frequency region around 100 kHz due to the in-service operation effect on the winding impedances [9]. The age of the transformer under study is 7 years old. The mutual inductance between the HV and LV windings is neglected in the simulation to reduce the model complexity. Additionally, the eddy current and hysteresis losses that appear at the frequency higher than 10 kHz are neglected due to the lower depth of the flux penetration into the core [1]. Anti-resonance in simulated frequency response by MTL model exists at 290 kHz while for measured frequency response, it appears at 230 kHz. Anti-resonance occurs due to either high total impedance of the winding at certain frequencies or non-consideration of the core flux linkage. The highest differences between simulated frequency response by MTL model and measured frequency response occur at the mid frequency region based on ASLE and RMSE can be seen in Table 4. At the low frequency region, the error based on ASLE analysis is 2.3427 for low frequency region and it increases at mid frequency region to 4.8535. The error slightly decreases to 1.7340 at the high frequency region for ASLE analysis. On the other hand, the highest error is 0.15823 at the mid frequency region followed by the low and high frequency regions based on RMSE.

**Table 4:** ASLE and RMSE errors for comparison of simulated frequency response based on MTL model and measurement.

Comparison	Frequency range	ASLE	RMSE
Measured—MTL	Low 20 Hz–1 kHz	2.3427	0.0999
	Mid 1 kHz–230 kHz	4.8535	0.1583
	High 230 kHz–2 MHz	1.7340	0.0802

### B. Comparison between frequency response based on FEM model and measurement

**Fig 12:**
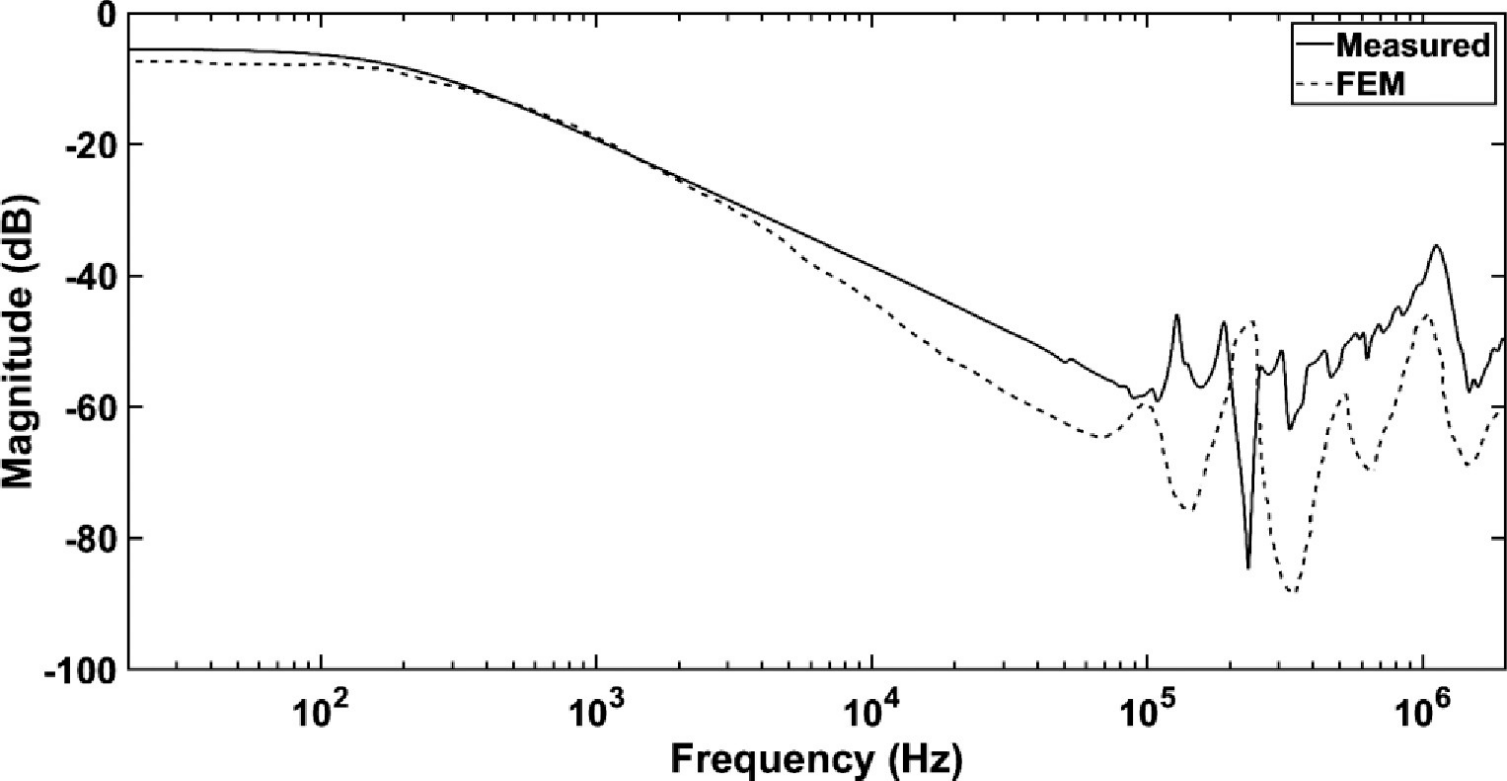
The comparison of simulated frequency response based on FEM model and measurement.

It is apparent that overall FEM model performs better than MTL model at the low and mid frequency regions whereby the simulated frequency response is closer to measured frequency response as shown in Fig 12. However, the occurrence of anti-resonance for simulated frequency response by FEM is farther than measured frequency response whereby it exists at 350 kHz. Due to the complexity involved in the geometrical construction, simplification by reduction and disconnection of the discs of the model has been carried out which results in deviation of frequency responses especially at the high frequency region. Additionally, the effect of mutual coupling between the HV and LV windings are not considered in the FEM model. These assumptions could lead to the differences of the frequency responses. Nevertheless, the differences between simulated frequency response by FEM model and measured frequency response is lower than MTL model based on RMSE and ASLE analyses at low and mid frequency region as shown in Table 5. Furthermore, the pattern of error increment in 3 frequency regions for FEM model is different than MTL model whereby the highest error is 2.6573 which is observed at the high frequency region followed by low and mid frequency regions based on ASLE. The same pattern is observed for analysis based on RMSE.

**Table 5:** ASLE and RMSE errors for comparison of simulated frequency response based on FEM model and measurement.

Comparison	Frequency range	ASLE	RMSE
Measured—FEM	Low 20 Hz–1 kHz	2.2653	0.1084
	Mid 1 kHz–230 kHz	0.8976	0.0203
	High 230 kHz–2 MHz	2.6573	0.1689

## Discussion

It is apparent the frequency responses modeled by FEM model could represent the measured frequency response better than MTL model except in the high frequency region as shown in Figs 11 and 12. Generally, in the mid frequency region between 1 kHz to 230 kHz, the frequency responses decrease for measured, MTL and FEM models. This phenomenon could be either due to the interaction of magnetizing inductance with the shunt capacitance of the winding or dominance of the leakage inductance in the mid frequency region. The error analyses reveal that both RMSE and ASLE are quite sufficient determine the differences in frequency responses obtained based on both MTL and FEM models in comparison with the measured frequency response. At the low frequency region from 20 Hz to 1 kHz, the simulated frequency response based on FEM model is closer to measured frequency response as compared to MTL model based on ASLE with errors of 2.2653 and 2.3427. The errors by RMSE are lower than ASLE with 0.1084 and 0.0999 due to the fact that the method is less sensitive towards horizontal changes, which is the case for the low frequency region responses [57]. FEM model could represent the measured frequency response at the mid frequency region from 1 kHz to 230 kHz better than MTL model based on ASLE and RMSE with errors of 0.8976 and 0.023. Meanwhile, at the high frequency region from 230 kHz to 2 MHz, MTL model represents the measured frequency response quite well as compared to FEM model with errors of 1.7340 and 0.0802 based on ASLE and RMSE. It could be due to the flux penetration to the core especially at frequency higher than 1 MHz [58]. This affects the inductance due to the residual flux present in the core [27]. The resonances in the FEM model overshoots in the high frequency region possibly due to the previous assumption of neglecting the effects of core at frequency range higher than 1 MHz. Such resonances and anti-resonances in the high frequency region of the FEM model is due to the high impedance matrix in the form of Z = R + jωL [27]. Each disc of the FEM model consists of 6 conductors and 8 discs generating 48 × 48 matrices. The discs are not connected during RLC parameter calculation resulting in higher matrix size. As a result, the AC resistances increase and affect the frequency response at the high frequency region [59]. Although some of the resonances are not exactly aligned at the same frequency, the overall shape of FEM simulated frequency response fairly in-line with the measured response. In addition, there is a possibility that the high frequency region might be influenced by transformer test setup during the measurement of FRA [60].

The differences between simulated frequency response by FEM model and measured frequency response could be due to various factors such as modeling simplification of the winding geometry and the effect of the clamping structure is neglected. Since the transformer under study has been in-service, the pressure of clamping structure might be slightly reduced which could affect the measured frequency response as reported in [52]. For MTL model, assumptions such as the non-consideration of insulation thickness in the terminal winding lead, mutual inductance between adjacent windings and the hysteresis/eddy current losses in the core could lead to deviation of the frequency response as compared to measurement. Furthermore, the spacers are neglected for the simulated frequency responses based on MTL and FEM models. The pressure by the spacers could increase the relative permittivity and lead to the increment of inter-disc capacitances [61].

The simulated frequency response by MTL model is quite simplified with less resonance and anti-resonance as compared to FEM model. Considering the stated factors, it can be implied that FEM model is more suitable to represent the measured frequency response as compared to MTL model especially at the low and mid frequency ranges. Based on both ASLE and RMSE, it is shown that apparent deviations with measured frequency response for FEM model only exists at the high frequency region.

## Conclusions

Overall, the proposed FEM model could be used to represent measured frequency response better than MTL model at low and mid frequency regions based on frequency plot and error analysis. At low frequency region, the FEM model performs almost similar to MTL model on representing the measured frequency response. At mid frequency region, FEM model performs well whereby a few of the resonance and anti-resonance of the measured frequency region could be modeled. The underestimation of the FEM model at high frequency region is due to large impedance matrix originated from 3-D FEM model which could be further investigated in the future study. Through Ansys Q3D and Simplorer circuit, the simplification of FEM model could be carried out with reasonable accuracy. The proposed model can be used for overvoltage studies of the transformer windings in each turn and disc with more accuracy. The application of the proposed FEM model can be extended to accurately model the transformer winding, thereby various types of winding deformations and displacements can be simulated and examined.
